# Impulsive Reaction-Diffusion Delayed Models in Biology: Integral Manifolds Approach

**DOI:** 10.3390/e23121631

**Published:** 2021-12-03

**Authors:** Gani Stamov, Ivanka Stamova, Cvetelina Spirova

**Affiliations:** 1 Department of Mathematical Physics, Technical University of Sofia, 8800 Sliven, Bulgaria; stamov@tu-sofia.bg (G.S.); cvetelina2802@abv.bg (C.S.); 2 Department of Mathematics, University of Texas at San Antonio, San Antonio, TX 78249, USA

**Keywords:** reaction-diffusion equations, delays, impulses, integral manifolds, stability

## Abstract

In this paper we study an impulsive delayed reaction-diffusion model applied in biology. The introduced model generalizes existing reaction-diffusion delayed epidemic models to the impulsive case. The integral manifolds notion has been introduced to the model under consideration. This notion extends the single state notion and has important applications in the study of multi-stable systems. By means of an extension of the Lyapunov method integral manifolds’ existence, results are established. Based on the Lyapunov functions technique combined with a Poincarè-type inequality qualitative criteria related to boundedness, permanence, and stability of the integral manifolds are also presented. The application of the proposed impulsive control model is closely related to a most important problems in the mathematical biology—the problem of optimal control of epidemic models. The considered impulsive effects can be used by epidemiologists as a very effective therapy control strategy. In addition, since the integral manifolds approach is relevant in various contexts, our results can be applied in the qualitative investigations of many problems in the epidemiology of diverse interest.

## 1. Introduction

The dynamics of reaction-diffusion and related equations are traditionally applied to problems in biology, population dynamics, ecology, and neurosciences [1,2,3,4]. Numerous reaction-diffusion models have also been proposed in epidemiology and virology [5,6,7,8,9,10]. In fact, reaction-diffusion terms in a model can adequately describe the time and spatial evolution, which is why reaction-diffusion epidemic models are still a very active area of research [11,12,13].

In addition, the effects of time delays on the dynamical properties of reaction-diffusion epidemic and virus dynamic models are well investigated [14,15,16,17]. It is well known that models that include delays have been introduced to account for the time between viral entry into a target cell and the production of new virus particles. For example, the work [18] studied the effect of the intracellular delay and the spatial diffusion of free virus on the global dynamics of a virus dynamics model using the following delayed reaction–diffusion system:

(1) $$\left\{\begin{array}{c} \frac{\partial T(x,t)}{\partial t}=-dT\left(x,t\right)-\frac{\beta V(x,t)T(x,t)}{1+\alpha_{1}T\left(x,t\right)+\alpha_{2}V\left(x,t\right)}+\lambda ,\\ \frac{\partial I(x,t)}{\partial t}=-aI\left(x,t\right)+e^{-a\tau }\frac{\beta V(x,t-\tau )T(x,t-\tau )}{1+\alpha_{1}T\left(x,t-\tau \right)+\alpha_{2}V\left(x,t-\tau \right)},\\ \frac{\partial V(x,t)}{\partial t}=d_{V}\Delta V-mV\left(x,t\right)+kI\left(x,t\right), \end{array}\right.$$

where t≥0, $x\in R^{3}$, *T*, *I* and *V* represent the susceptible cells, infected cells, and free viruses, respectively, λ, dT and βVT are the production rate, the death rate, and the infection by the virus rate of the susceptible cells, respectively, aI is the death rate of the infected cells, kI and mV are, respectively, the production from the infected cells and the decay rates, ΔV is the Laplace operator for *V*, $d_{V}$ is the diffusion coefficient, the ratio

$$\frac{\beta V(x,t)T(x,t)}{1+\alpha_{1}T\left(x,t\right)+\alpha_{2}V\left(x,t\right)},$$

where $\alpha_{1}$ and $\alpha_{2}$ are constant represents Beddington–DeAngelis functional response of infection rate, $e^{-a\tau }$ denotes the surviving rate of infected cells before it becomes productively infected, τ is a positive constant that represents the delay.

The model (1) is a generalization of the delayed virus dynamics model with the Beddington–DeAngelis functional response proposed in [19], where the authors assumed that the generation of virus producing cells at time *t* is due to the infection of target cells at time t−τ without considering diffusion effects. However, the spatial effects cannot be neglected in studying the spread of epidemics. When consider the reaction-diffusion case [18], the delay τ has an intracellular origin. In fact, the necessity of considering delays in epidemiology models is biologically reasonable since the infectious processes are not instantaneous. For example, for $\alpha_{1}=0$ the model (1) is reduced to the HBV model with the diffusion and time delay that has been investigated in [9], where time delay representing the intracellular incubation period, i.e., the lag between the time a cell becomes infected and when it begins to infect other cells on the global dynamics of an HBV model with saturation response of the infection rate. The paper [9] also proposes a very good overview of some delayed models in epidemiology and the origin of the delay terms in such models.. Note that the dynamic behaviors of such models have important relationships with the entropy phenomena in biological and virology systems [20,21].

In their efforts to propose effective drug therapies, many authors considered impulsive vaccination and active impulsive drug-treatment strategies [22,23,24,25,26]. Such therapies can be regarded as impulsive effects and can be accurately described by impulsive differential equations. Indeed, mathematical models using impulsive differential equations have got a lot of attention in the treatment policies of many diseases. For some excellent fundamental and qualitative results on impulsive differential systems, we refer to [27,28,29,30,31].

In addition, the specific control strategy, called "impulsive control" also plays a major role in many applied problems. The main advantages of such a strategy lie in the fact that it is applied just in some discrete times and can reduce the control cost and the amount of transmitted information drastically [32,33,34,35,36,37,38].

Impulsive control therapeutic strategy has been proposed to some very recent epidemic models of great importance for modern society. For example, in [39] the authors applied impulsive controls to a SQEIAR model to reduce the size of susceptible, infected, exposed, and asymptomatic groups to consequently eradicate the infection. A special attention is devoted to the COVID-19 pandemic case. An impulsive control approach has been proposed in [40] to determine the effect of DAA therapy in hepatitis C treatment. The effectiveness of impulse control, which has a certain enlightening effect on the actual epidemic prevention work for reaction-diffusion ecosystems has been demonstrated in [41]. The topology of stable periodic solutions of an SIR epidemic dynamics model with impulsive vaccination control has been reported in [42]. In [43] an impulsive vaccination strategy is proposed in the study of the global dynamics and bifurcation phenomena of state-dependent impulsive SIR models. Hence, impulsive control methodology is very effective and impulsive mathematical models may offer a convenient method for rationally designing therapy based on the properties of single agents [40]. However, to the best of our knowledge, an impulsive control approach has not been applied to the model (1) proposed in [18], and one of the main aims of this research is to fill the gap.

Most of the reported results for impulsive epidemic models are related to stability results [22,23,25,26,41,42,43]. Indeed, stability of the states is a qualitative property of a crucial importance for the systems. However, in the existing results for impulsive models in biology and epidemiology, the authors investigated the qualitative behavior of a single state of interest: unique stationary state (unique equilibrium point), periodic state, and semi-trivial periodic state.

In the proposed paper, the application of the impulsive control approach to the model (1) considered in [18] is applied. The purpose of the paper and one of the main research questions is to investigate the effect of the impulses on the qualitative behavior of the states and to show how by means of appropriate impulsive perturbations we can control the stability behavior of the system.

We also introduce the concept of integral manifolds to the extended impulsive model. The existence and stability of integral manifolds notions are more general than the corresponding notions applied to single states. The method of integral manifolds is widely accepted as a useful tool in the qualitative analysis of systems with piece-wise constant arguments [44], impulsive systems [45], numerous systems in nonlinear mechanics [46], partial differential equations [47], impulsive differential systems defined on torus [48], systems with nonlinear oscillations [49], uncertain systems with variable impulsive perturbations [50], and fractional models [51]. Therefore, the connection of this method to models in science, engineering, and biology is highly important in terms of theoretical developments and practical applications. However, the method of integral manifolds is not yet applied to reaction-diffusion models in epidemiology, which is the main goal and contribution of the present research.

The main novelty of the paper is in five points:

(1) An impulsive control strategy is considered for a class of delayed reaction-diffusion models in biology, which arises naturally in a wide variety of biological applications and allows for synchronization of a complex system by using only small control impulses, even though the system’s behavior may follow unpredictable patterns;
(2) The integral manifold notion is introduced for the first time for the model under consideration, which generalizes the single state concepts and is very effective in systems with several equilibria;
(3) New existence, boundedness, and permanence results are established with respect to integral manifolds;
(4) Criteria for asymptotic stability of an integral manifold related to the impulsive model under consideration are also proved;
(5) We apply a Poincarè-type integral inequality, which allows for more accurate estimation of the reaction diffusion terms, and leads to a better exploration of the diffusion effect.

Since delayed reaction-diffusion models remain very popular in science, biology, engineering, neurocomputing, etc., analyzing their qualitative behavior would have diverse applications. Hence, the developed extended integral manifolds qualitative results will be of a great importance not only for researchers in applied mathematics and mathematical biology, but also for a wide audience of professionals all over the world.

The construction of the rest of the manuscript is as follows. In Section 2 we propose the class of impulsive delayed reaction-diffusion epidemic models. The concept of integral manifolds is also introduced for the model under consideration. In addition, notations, definitions, and preliminary results are presented. Section 3 is devoted to existence, boundedness, and permanence results for integral manifolds associated with those introduced in the Section 2 model. In Section 4, new sufficient conditions for the uniform global asymptotic stability of integral manifolds are established. Finally, some conclusions and directions for future research are stated in Section 5.

## 2. Model Description and Preliminaries

In this section, we extend and improve some recent results in the existing literature [9,18,19] and propose an impulsive generalization of the model (1). To this end, we will use the following notations: $\left||x||=\right|x_{1}\left|+\right|x_{2}\left|+\right|x_{3}|$ will be the norm of $x=\left(x_{1},x_{2},x_{3}\right)\in R^{3}$, $R_{+}=\left[0,\infty \right)$. Additionally, in $R^{3}$ we will consider an open and bounded set Ω that has smooth boundary ∂Ω and the measure is denoted by mesΩ>0. Let the zero 0=(0,0,0)∈Ω.

We introduce, in this paper, an impulsive delayed reaction-diffusion epidemic model in the following form:

(2) $$\left\{\begin{array}{c} \frac{\partial T(x,t)}{\partial t}=-dT\left(x,t\right)-\frac{\beta V(x,t)T(x,t)}{1+\alpha_{1}T\left(x,t\right)+\alpha_{2}V\left(x,t\right)}+\lambda ,\;t\neq t_{i},\\ \frac{\partial I(x,t)}{\partial t}=-aI\left(x,t\right)+e^{-a\tau }\frac{\beta V(x,t-\tau )T(x,t-\tau )}{1+\alpha_{1}T\left(x,t-\tau \right)+\alpha_{2}V\left(x,t-\tau \right)},\;t\neq t_{i},\\ \frac{\partial V(x,t)}{\partial t}=d_{V}\Delta V-mV\left(x,t\right)+kI\left(x,t\right),\;t\neq t_{i},\\ T\left(x,t_{i}^{+}\right)=T\left(x,t_{i}\right)+P_{1i}\left(T\left(x,t_{i}\right)\right),\\ I\left(x,t_{i}^{+}\right)=I\left(x,t_{i}\right)+P_{2i}\left(I\left(x,t_{i}\right)\right),\\ V\left(x,t_{i}^{+}\right)=V\left(x,t_{i}\right)+P_{3i}\left(V\left(x,t_{i}\right)\right),\;\;i=1,2,\ldots , \end{array}\right.$$

where x∈Ω, $t\geq t_{0}$, $t_{0}\in R_{+}$:

(i) The impulsive instants $t_{i}$ are such that $0<t_{1}<t_{2}<\cdots <t_{i}<t_{i+1}<$ and $\lim_{i\to \infty }t_{i}=\infty$;
(ii) All parameters in the first three equations have the same meaning as in (1) for $t\neq t_{i}$, i=1,2,…;
(iii) Functions $P_{ji}$, j=1,2,3, i=1,2,… are real and determine the controlled outputs $T\left(.,t^{+}\right),\;I\left(.,t^{+}\right),\;V\left(.,t^{+}\right)$ at times $t_{i}$, i=1,2,….

The model (2) generalizes and extends some existing models investigated in [9,18,19] to the impulsive case. Adding the *impulsive control* equations from fourth to sixth to the model (1) allows the application of the impulsive control approach to models of type (1). In fact, the impulsive functions $P_{ji}$, j=1,2,3, i=1,2,… can be designed as efficient impulsive controllers.

Note that, the function

$$f\left(T,V\right)=\frac{V\;T}{1+\alpha_{1}T+\alpha_{2}V}$$

is Lipschitz [18], and let L>0 denotes the Lipschitz constant for this function.

The initial and boundary values for the model (2) are set as

(3) $$T\left(x,t\right)=0,\;\;I\left(x,t\right)=0,\;V\left(x,t\right)=0,\;t\in \left[t_{0}-\tau ,\infty \right),\;x\in \partial \Omega ,$$

(4) $$\left\{\begin{array}{c} T\left(x,t-t_{0}\right)=\phi_{0T}\left(x,t-t_{0}\right)\geq 0,\\ I\left(x,t-t_{0}\right)=\phi_{0I}\left(x,t-t_{0}\right)\geq 0,\\ V\left(x,t-t_{0}\right)=\phi_{0V}\left(x,t-t_{0}\right)\geq 0,\;x\in \Omega ,\;t\in \left[t_{0}-\tau ,t_{0}\right], \end{array}\right.$$

where $\phi_{0T}\left(x,\xi \right)$, $\phi_{0I}\left(x,\xi \right)$, $\phi_{0V}\left(x,\xi \right)$ are nonnegative real-valued functions well defined on Ω×[−τ,0], bounded and piecewise continuous with respect to ξ with a possibly finite number of discontinuity points of the first kind ξ∈[−τ,0] such that $\phi_{0T}\left(x,\xi^{+}\right)$, $\phi_{0T}\left(x,\xi^{-}\right)$, $\phi_{0I}\left(x,\xi^{+}\right)$, $\phi_{0I}\left(x,\xi^{-}\right)$, $\phi_{0V}\left(x,\xi^{+}\right)$, $\phi_{0V}\left(x,\xi^{-}\right)$ exist and are nonnegative, and $\phi_{0T}\left(x,\xi^{-}\right)=\phi_{0T}\left(x,\xi \right)$, $\phi_{0I}\left(x,\xi^{-}\right)=\phi_{0I}\left(x,\xi \right)$, $\phi_{0V}\left(x,\xi^{-}\right)=\phi_{0V}\left(x,\xi \right)$. The class of all functions $\phi_{0}=\left(\phi_{0T}\left(x,\xi \right),\;\phi_{0I}\left(x,\xi \right),\;\phi_{0V}\left(x,\xi \right)\right)$ with such components will be denoted by PC, and by PCB we will denote the class of all bounded functions $\phi_{0}\in \mathrm{PC}$.

It is assumed that d,β,a,k,m are positive constant and the motion of virus follows the Fickan diffusion [52,53].

To simplify the notations, we will denote by $U\left(x,t\right)=U\left(x,t;\phi_{0}\right),\;U=\left(T,I,V\right)$ any solution of (2) with initial function $\phi_{0}=\left(\phi_{0T}\left(x,\xi \right),\;\phi_{0I}\left(x,\xi \right),\;\phi_{0V}\left(x,\xi \right)\right)=(T_{0}\left(x,\xi \right),\;I_{0}\left(x,\xi \right),$ $V_{0}\left(x,\xi \right))\in \mathrm{PC}$. The norm of U=(T,I,V) for $t\geq t_{0}$ will be defined as

$${\left||U\left(x,t\right)|\right|}_{2}={\left[\int_{\Omega }\left(T^{2}\left(x,t\right)+I^{2}\left(x,t\right)+V^{2}\left(x,t\right)\right)dx\right]}^{1/2}$$

and the norm of the function ϕ∈PC is denoted as ${\left||.|\right|}_{\tau }$ and is defined by ${\left||\phi |\right|}_{\tau }=\sup_{-\tau \leq \xi \leq 0}{\left||\phi \left(x,\xi \right)|\right|}_{2}.$

It is well known [27,28,29,30,31] that the solutions of the impulsive initial-value boundary problems (2)–(4)

$$U\left(x,t;\phi_{0}\right)=\left(T\left(x,t;\phi_{0}\right),I\left(x,t;\phi_{0}\right),V\left(x,t;\phi_{0}\right)\right)$$

are piecewise continuous functions with first kind points of discontinuity at which they are continuous from the left.

For more results on the corresponding fundamental and qualitative dynamical behaviors of impulsive reaction-diffusion systems, we refer to [36,54,55,56,57]. The consideration of impulsive conditions allows us to take into account short-term effects on susceptible cells, infected cells, and free viruses.

The paper [18] discussed the global asymptotic stability of an infected equilibrium of the model (1), which generalizes several important models in biology. In this paper, we will develop these results further, and we will introduce the integral manifolds approach to the impulsive model (2) [44,45,46,47,48,49,50,51]. Let $\Theta =\{U\in R^{3}:T\geq 0,\;I\geq 0,\;V\geq 0\}$ and we assume that $T(x,t_{i}^{+})\geq 0$, $I(x,t_{i}^{+})\geq 0$, $V(x,t_{i}^{+})\geq 0$ for any x∈Ω and i=1,2,…. In what follows, we restrict our attention only to those solutions *U* which evolve in the space Θ which is natural from a biological perspective [58].

**Definition** **1.** *An arbitrary manifold M in the space $\Omega \times [t_{0}-\tau ,\infty )\times \Theta$ is called an integral* manifold *of the model (2), if for any state $U\left(x,t\right)=U\left(x,t;\phi_{0}\right)$ of (2) such that $(x,\xi ,\phi_{0}\left(x,\xi \right))\in M$, (x,ξ)∈Ω×[−τ,0] we have (x,t,U(x,t))∈M, x∈Ω and $t\geq t_{0}$.*

We will also introduce the following notations related to a manifold M⊂Ω×[−τ,∞)×Θ:

$M\left(x,t\right)=\left\{U\in \Theta :\left(x,t,U\right)\in M,\left(x,t\right)\in \Omega \times \left[t_{0},\infty \right)\right\}$;

$M_{0}\left(x,t\right)=\left\{Z\in \Theta :\;\left(x,t,Z\right)\in M,\;\left(x,t\right)\in \Omega \times \left[t_{0}-\tau ,t_{0}\right]\right\}$;

$D\left(U,M\left(x,t\right)\right)=\underset{\tilde{U}\in M\left(x,t\right)}{\inf}||U-\tilde{U}{\left|\right|}_{2}$ is the distance between U∈Θ and M(x,t);

$M\left(x,t\right)\left(\varepsilon \right)=\left\{U\in \Theta :\;D(U,M(x,t))<\varepsilon \right\}\;\left(\varepsilon >0\right)$ is an ε- neighborhood of M(x,t);

$D_{0}\left(\phi ,M_{0}\left(x,t\right)\right)=\underset{\xi \in [-\tau ,0]}{\sup}D\left(\phi \left(x,\xi \right),M_{0}\left(x,\xi \right)\right),\;\phi \in \mathrm{PC}$;

$M_{0}\left(x,t\right)\left(\varepsilon \right)=\left\{\phi \in \mathrm{PC}:\;D_{0}\left(\phi ,M_{0}\left(x,t\right)\right)<\varepsilon \right\}$ is an ε- neighborhood of $M_{0}\left(x,t\right)$;

$\bar{M}\left(x,t\right)\left(\varepsilon \right)=\left\{U\in \Theta :\;D(U,M(x,t))\leq \varepsilon \right\}$;

$\bar{M_{0}}\left(x,t\right)\left(\varepsilon \right)=\left\{\phi \in \mathrm{PC}:\;D_{0}\left(\phi ,M_{0}\left(x,t\right)\right)\leq \varepsilon \right\}$;

$\bar{S_{\alpha }}={\left\{U\in \Theta :\;||U|\right|}_{2}\leq \alpha \}$, α>0;

$\bar{S_{\alpha }}\left(\mathrm{PC}\right)={\left\{\phi \in \mathrm{PC}:\;||\phi |\right|}_{\tau }\leq \alpha \}$.

Throughout this paper, we will also assume that the next assumptions hold:

A1. The impulsive functions $P_{ji}$ are continuous and $P_{ji}\left(0\right)=0$ for all j=1,2,3 and i=1,2,….

A2. The set M(x,t) is nonempty for $\left(x,t\right)\in \Omega \times \left[t_{0},\infty \right)$.

A3. The set $M_{0}\left(x,t\right)$ is nonempty for $\left(x,t\right)\in \Omega \times \left[t_{0}-\tau ,t_{0}\right]$.

It is well known that boundedness and permanence are two qualitative properties of a significant importance for models in biology [58,59,60]. In this paper, the concepts will be generalized to the integral manifolds method as follows.

**Definition** **2.** *An integral manifold $M\subset \Omega \times [t_{0}-\tau ,\infty )\times \Theta$ of system (2) is:*

*(a)*  *Equi-M-bounded, if for any η>0, α>0, $t_{0}\in R_{+}$ there exists a constant $b=b(\eta ,\alpha ,t_{0})>0$ such that for each $\phi_{0}\in \bar{S_{\alpha }}\left(\mathrm{PC}\right)\cap \bar{M_{0}}\left(x,\xi \right)\left(\eta \right)$, we have $U\left(x,t;\phi_{0}\right)\in M\left(x,t\right)\left(b\right)$ for x∈Ω, $t\geq t_{0}$;*
*(b)*  *Uniformly-M-bounded, if b in (a) is independent of $t_{0}\in R_{+}$;*
*(c)*  *Quasi-ultimately-M-bounded, if there exists B>0 such that for any η>0, α>0, $t_{0}\in R_{+}$ there exists a constant $T=T(\eta ,\alpha ,t_{0})>0$ such that for each $\phi_{0}\in \bar{S_{\alpha }}\left(\mathrm{PC}\right)\cap \bar{M_{0}}\left(x,\xi \right)\left(\eta \right)$, we have $U\left(x,t;\phi_{0}\right)\in M\left(x,t\right)\left(B\right)$ for x∈Ω, $t\geq t_{0}+T$;*
*(d)*  *Quasi-uniformly-ultimately-M-bounded, if T in (c) is independent of $t_{0}\in R_{+}$;*
*(e)*  *Ultimately-M-bounded, if both (a) and (c) hold;*
*(f)*  *Uniformly-ultimately-M-bounded, if both (b) and (d) hold.*

**Definition** **3.** *An integral manifold $M\subset \Omega \times [t_{0}-\tau ,\infty )\times \Theta$ of system (2) is:*

*(a)*  *Quasi-M-permanent, if for each $\phi_{0}\in \mathrm{PC}$ and any $t_{0}\in R_{+}$ there exist constants $b_{1}=b_{1}\left(\phi_{0},t_{0}\right)>0$ and $b_{2}=b_{2}\left(\phi_{0},t_{0}\right)>0$ such that $b_{1}\leq T\left(x,t\right)\leq b_{2}$, $b_{1}\leq I\left(x,t\right)\leq b_{2}$, $b_{1}\leq V\left(x,t\right)\leq b_{2}$ for x∈Ω, $t\geq t_{0}$, where U=(T,I,V)∈M(x,t);*
*(b)*  *Uniformly quasi-M-permanent, if the constants $b_{1}$ and $b_{2}$ in (a) are independent of $t_{0}\in R_{+}$;*
*(c)*  *M-permanent if it is quasi-M-permanent and if there exist constants B≥b>0 such that for each $\phi_{0}\in \mathrm{PC}$ and any $t_{0}\in R_{+}$ there exists a constant $T=T(\phi_{0},t_{0})>0$ such that b≤T(x,t)≤B, b≤I(x,t)≤B, b≤V(x,t)≤B for x∈Ω, $t\geq t_{0}+T$, where U=(T,I,V)∈M(x,t);*
*(d)*  *Uniformly M-permanent if it is uniformly quasi-M-permanent and there exist constants B≥b>0 such that for each $\phi_{0}\in \mathrm{PC}$ there exists a constant $T=T(\phi_{0})>0$ such that if $t_{0}\in R_{+}$, then b≤T(x,t)≤B, b≤I(x,t)≤B, b≤V(x,t)≤B for x∈Ω, $t\geq t_{0}+T$, where U=(T,I,V)∈M(x,t).*

The extensions of some stability concepts [18] to the integral manifolds case are as follows.

**Definition** **4.** *An integral manifold M of system (2) is:*

*(a)*  *Stable, if for any ε>0, α>0, $t_{0}\in R_{+}$ there exists a $\delta =\delta (\alpha ,t_{0},\varepsilon )>0$ such that for each $\phi_{0}\in \bar{S_{\alpha }}\left(\mathrm{PC}\right)\cap \bar{M_{0}}\left(x,\xi \right)\left(\delta \right)$, we have $U\left(x,t;\phi_{0}\right)\in M\left(x,t\right)\left(\varepsilon \right)$ for x∈Ω and $t\geq t_{0}$;*
*(b)*  *Uniformly stable, if the number δ from (a) depends only on ε>0;*
*(c)*  *Uniformly globally asymptotically stable, if it is a uniformly stable, uniformly-M-bounded, and for any ε>0 and η>0 there exists a T=T(η,ε)>0 such that for any $t_{0}\in R_{+}$, α>0 and each $\phi_{0}\in \bar{S_{\alpha }}\left(\mathrm{PC}\right)\cap \bar{M_{0}}\left(x,\xi \right)\left(\eta \right)$, we have $U\left(x,t;\phi_{0}\right)\in M\left(x,t\right)\left(\varepsilon \right)$ for x∈Ω, $t\geq t_{0}+T$.*

Next, we will define the class of Lyapunov-like functions [31,36,55,56] that will be used in the study of the qualitative properties of integral manifolds, related to the model (2).

Denote $t_{0}=0$ and consider the sets:

$$G_{i}=\left\{\left(U,t\right):\;t_{i-1}<t<t_{i},\;U=\left(T,I,V\right)\in \Theta \right\},\;i=1,2,\ldots ,\;\;G=\overset{\infty }{\underset{i=1}{⋃}}G_{i}.$$

**Definition** **5.** *A Lyapunov-type function $W:\Theta \times R_{+}\to R_{+}$ belongs to the class $W_{M}$ if:*

*1*. *W(U,t) is continuous in G, locally Lipschitz continuous with respect to its first argument on each of the sets $G_{i}$, and W(U(x,t),t)=0 for (x,t,U)∈M, t≥0, W(U(x,t),t)>0 for $\left(x,t,U\right)\in \left\{\Omega \times R_{+}\times \Theta \right\}\setminus M$.*
*2*. *$W(U,t_{i}^{-})$ and $W(U,t_{i}^{+})$ exist for each i=1,2,…, and $W\left(U,t_{i}^{-}\right)=W\left(U,t_{i}\right)$.*

Let $t\in R_{+}$, $t\neq t_{i}$, i=1,2,…, $\bar{\phi }\in \mathrm{PC}$, and the function $W\in W_{M}$. Then by $D^{+}W\left(\bar{\phi }\left(.,0\right),t\right)$ we will denote the upper right derivative of $W\in W_{M}$ defined as

$$D^{+}W\left(\bar{\phi }\left(.,0\right),t\right)=\lim_{\chi \to 0^{+}}\sup\frac{1}{\chi }\left[W(U\left(.,t+\chi ;\bar{\phi }\left(.,0\right),t+\chi \right)-W\left(\bar{\phi }\left(.,0\right),t\right)\right].$$

The proof of the next Lyapunov-Razumikhin-type result is similar to those of Theorem 1.21 in [31], and we will omit it.

**Lemma** **1.** *Assume that A1–A3 hold, and for $W\in W_{M}$, ϕ∈PC, $P_{i}=\left(P_{1i},P_{2i},P_{3i}\right)$ and $t\in R_{+}$*

$$W\left(\phi \left(.,0\right)+P_{i}\left(\phi ,.\right),t^{+}\right)\leq W\left(\phi \left(.,0\right),t\right),\;t=t_{i},\;i=1,2,\ldots ,$$

*the inequality*

$$D^{+}W\left(\phi \left(.,0\right),t\right)\leq \mu W\left(\phi \left(.,0\right),t\right),\;t\neq t_{i},\;\mu \in R$$

*is valid whenever*

W(ϕ(.,ξ),t+ξ)≤W(ϕ(.,0),t),−τ≤ξ≤0.

*Then*

$$W\left(U\left(.,t\right),t\right)\leq \underset{-\tau \leq \xi \leq 0}{\sup}W\left(\phi_{0}\left(.,\xi \right),0\right)e^{\mu t},\;t\geq 0.$$

For μ=0, we have the following comparison result.

**Corollary** **1.** *Assume that A1–A3 hold, and for $W\in W_{M}$, ϕ∈PC, $P_{i}=\left(P_{1i},P_{2i},P_{3i}\right)$ and $t\in R_{+}$*

$$W\left(\phi \left(.,0\right)+P_{i}\left(\phi ,.\right),t^{+}\right)\leq W\left(\phi \left(.,0\right),t\right),\;t=t_{i},\;i=1,2,\ldots ,$$

*the inequality*

$$D^{+}W\left(\phi \left(.,0\right),t\right)\leq 0,\;t\neq t_{i},\;\mu \in R$$

*is valid whenever*

W(.,ξ),t+ξ)≤W(ϕ(.,0),t),−τ≤ξ≤0.

*Then*

$$W\left(U\left(.,t\right),t\right)\leq \underset{-\tau \leq \xi \leq 0}{\sup}W\left(\phi_{0}\left(.,\xi \right),0\right),\;t\geq 0.$$

**Remark** **1.** *For more results on the Lyapunov–Razumikhin approach applied to different models represented as classes of differential equations, we refer the reader to* [29,30,31,36,55,56,61].

**Remark** **2.** *Note that, the Lyapunov function method is also widely used in the standard energy method. In addition, the entropy can be considered as a Lyapunov function for isolated systems* [62].

Finally, the following Poincarè-type integral inequality [63] will be used in our qualitative analysis. Let $\Omega =\prod_{q=1}^{n}\left[a_{j},b_{j}\right]$, $a_{j},\;b_{j}=const\in R$, j=1,2,3 and $\Lambda =\max\{b_{j}-a_{j},\;j=1,2,3\}$.

**Lemma** **2.** [63,64] *For any real-valued function w(x) that belongs to $C^{1}\left(\Omega \right)$ the following relation is valid*

$$\int_{\Omega }w^{2}\left(x\right)dx\leq \frac{\Lambda^{2}}{12}\int_{\Omega }{\left|\nabla w\left(x\right)\right|}^{2}dx.$$

## 3. Existence, Boundedness, and Permanence of Integral Manifolds

We will consider the set Ω of points *x*, $x=(x_{1},x_{2},x_{3})$ defined as $a_{j}\leq \left|x_{j}\right|\leq b_{j}$, where $a_{j},b_{j}=const>0$, or $\Omega =\prod_{j=1}^{3}\left[a_{j},b_{j}\right]$.

**Theorem** **1.** *Assume that:*

*1*. *Assumptions A1–A3 are satisfied.*
*2*. *$M\subset \Omega \times [t_{0}-\tau ,\infty )\times \Theta$ is a manifold in the extended phase space of the model (2).*
*3*. *The functions $P_{ji}$ are such that*
$$P_{1i}\left(T\left(x,t_{i}\right)\right)=-\gamma_{1i}T\left(x,t_{i}\right),\;P_{2i}\left(I\left(x,t_{i}\right)\right)=-\gamma_{2i}I\left(x,t_{i}\right),\;P_{3i}\left(V\left(x,t_{i}\right)\right)=-\gamma_{3i}V\left(x,t_{i}\right),$$
$$\;0<\gamma_{ji}<2,\;\;j=1,2,3,\;i=1,2,\ldots .$$
*4*. *For the model’s parameters the following conditions hold*
$$\frac{3L\beta e^{-a\tau }}{2}<d,\;\;L\beta e^{-a\tau }+\frac{1}{2}k<a,\;\;\frac{1}{2}\left(L\beta e^{-a\tau }+k\right)<m+\frac{12d_{V}}{\Lambda^{2}},$$
$$\mu =\max\left\{\frac{3}{2}L\beta e^{-a\tau }-d,\;L\beta e^{-a\tau }-a+\frac{1}{2}k,\;\frac{1}{2}\left(L\beta e^{-a\tau }+k\right)-m-\frac{12d_{V}}{\Lambda^{2}}\right\}$$
$$+\frac{1}{2}\;L\beta e^{-a\tau }\leq 0.$$
*Then M is an integral manifold of the model (2).*

**Proof.**  Let $U\left(x,t\right)=U\left(x,t;\phi_{0}\right),\;U=\left(T,I,V\right)$ be a solution of the initial value boundary problems (2)–(4) for x∈Ω, $\phi_{0}\in \mathrm{PCB}$. According to A2, there exists at least one solution of (2) $\tilde{U}\left(x,t\right)=\left(\tilde{T}\left(x,t\right),\tilde{I}\left(x,t\right),\tilde{V}\left(x,t\right)\right)\in M\left(x,t\right)$.

We will use a Lyapunov function $W\in W_{M}$ defined as

$$W\left(U,t\right)=\frac{1}{2}D^{2}\left(U,M\left(x,t\right)\right)=\frac{1}{2}{\left(\underset{\tilde{U}\in M(x,t)}{\inf}||U-\tilde{U}{\left|\right|}_{2}\right)}^{2}$$

(5) $$=\underset{\tilde{U}\in M(x,t)}{\inf}\frac{1}{2}\int_{\Omega }\left({\left(T\left(x,t\right)-\tilde{T}\left(x,t\right)\right)}^{2}+{\left(I\left(x,t\right)-\tilde{I}\left(x,t\right)\right)}^{2}+{\left(V\left(x,t\right)-\tilde{V}\left(x,t\right)\right)}^{2}\right)dx.$$

For any $t=t_{i}$, i=1,2,…, condition 3 of Theorem 1 implies

$$\frac{1}{2}\int_{\Omega }{\left(1-\gamma_{1i}\right)}^{2}{\left(T\left(x,t\right)-\tilde{T}\left(x,t\right)\right)}^{2}dx<\frac{1}{2}\int_{\Omega }{\left(T\left(x,t\right)-\tilde{T}\left(x,t\right)\right)}^{2}dx,$$

$$\frac{1}{2}\int_{\Omega }{\left(1-\gamma_{2i}\right)}^{2}{\left(I\left(x,t\right)-\tilde{I}\left(x,t\right)\right)}^{2}dx<\frac{1}{2}\int_{\Omega }{\left(I\left(x,t\right)-\tilde{I}\left(x,t\right)\right)}^{2}dx,$$

$$\frac{1}{2}\int_{\Omega }{\left(1-\gamma_{3i}\right)}^{2}{\left(V\left(x,t\right)-\tilde{V}\left(x,t\right)\right)}^{2}dx<\frac{1}{2}\int_{\Omega }{\left(V\left(x,t\right)-\tilde{V}\left(x,t\right)\right)}^{2}dx,$$

and, hence, for any ϕ∈PC

(6) $$W\left(\phi \left(.,0\right)+P_{i}\left(\phi ,.\right),t^{+}\right)<W\left(\phi \left(.,0\right),t\right),\;t=t_{i},\;i=1,2,\ldots .$$

Then, for the derivative of the function W(U(.,t),t), for any $t\neq t_{i}$, i=1,2,…, it follows

$$\frac{dW(U(.,t),.)}{dt}$$

$$\leq \frac{1}{2}\int_{\Omega }\frac{d}{dt}\left({\left(T\left(x,t\right)-\tilde{T}\left(x,t\right)\right)}^{2}+{\left(I\left(x,t\right)-\tilde{I}\left(x,t\right)\right)}^{2}+{\left(V\left(x,t\right)-\tilde{V}\left(x,t\right)\right)}^{2}\right)dx$$

$$=\int_{\Omega }\left(T\left(x,t\right)-\tilde{T}\left(x,t\right)\right)\frac{\partial \left(T\left(x,t\right)-\tilde{T}\left(x,t\right)\right)}{\partial t}dx$$

$$+\int_{\Omega }\left(I\left(x,t\right)-\tilde{I}\left(x,t\right)\right)\frac{\partial \left(I\left(x,t\right)-\tilde{I}\left(x,t\right)\right)}{\partial t}dx$$

$$+\int_{\Omega }\left(V\left(x,t\right)-\tilde{V}\left(x,t\right)\right)\frac{\partial \left(V\left(x,t\right)-\tilde{V}\left(x,t\right)\right)}{\partial t}dx.$$

The fact that $\tilde{U}=\left(\tilde{T},\tilde{I},\tilde{V}\right)\in M\left(x,t\right)$ implies the following estimates:

$$\int_{\Omega }\left(T\left(x,t\right)-\tilde{T}\left(x,t\right)\right)\frac{\partial \left(T\left(x,t\right)-\tilde{T}\left(x,t\right)\right)}{\partial t}dx$$

$$=\int_{\Omega }\left(T\left(x,t\right)-\tilde{T}\left(x,t\right)\right)(-d(T\left(x,t\right)-\tilde{T}\left(x,t\right))-\frac{\beta V(x,t)T(x,t)}{1+\alpha_{1}T\left(x,t\right)+\alpha_{2}V\left(x,t\right)}$$

$$+\frac{\beta \tilde{V}\left(x,t\right)\tilde{T}\left(x,t\right)}{1+\alpha_{1}\tilde{T}\left(x,t\right)+\alpha_{2}\tilde{V}\left(x,t\right)})dx$$

(7) $$\leq \left(\frac{3L\beta e^{-a}}{2}-d\right)\int_{\Omega }{\left(T\left(x,t\right)-\tilde{T}\left(x,t\right)\right)}^{2}dx+\frac{1}{2}L\beta e^{-a}\int_{\Omega }{\left(V\left(x,t\right)-\tilde{V}\left(x,t\right)\right)}^{2}dx;$$

$$\int_{\Omega }\left(I\left(x,t\right)-\tilde{I}\left(x,t\right)\right)\frac{\partial \left(I\left(x,t\right)-\tilde{I}\left(x,t\right)\right)}{\partial t}dx$$

$$=\int_{\Omega }\left(I\left(x,t\right)-\tilde{I}\left(x,t\right)\right)(-a(I\left(x,t\right)-\tilde{I}\left(x,t\right))-e^{-a\tau }\frac{\beta V(x,t-\tau )T(x,t-\tau )}{1+\alpha_{1}T\left(x,t-\tau \right)+\alpha_{2}V\left(x,t-\tau \right)}$$

$$+e^{-a\tau }\frac{\beta \tilde{V}\left(x,t-\tau \right)\tilde{T}\left(x,t-\tau \right)}{1+\alpha_{1}\tilde{T}\left(x,t-\tau \right)+\alpha_{2}\tilde{V}\left(x,t-\tau \right)})dx$$

$$\leq \left(L\beta^{-a\tau }e-a\right)\int_{\Omega }{\left(I\left(x,t\right)-\tilde{I}\left(x,t\right)\right)}^{2}dx+\frac{1}{2}L\beta e^{-a\tau }\int_{\Omega }\underset{-\tau \leq \xi \leq 0}{\sup}{\left(V\left(x,\xi \right)-\tilde{V}\left(x,\xi \right)\right)}^{2}dx$$

(8) $$+\frac{1}{2}L\beta e^{-a\tau }\int_{\Omega }\underset{-\tau \leq \xi \leq 0}{\sup}{\left(T\left(x,\xi \right)-\tilde{T}\left(x,\xi \right)\right)}^{2}dx.$$

From the conditions of Theorem 1 and Lemma 2, by the Green’s theorem, we have

$$\int_{\Omega }\left(V\left(x,t\right)-\tilde{V}\left(x,t\right)\right)\frac{\partial \left(V\left(x,t\right)-\tilde{V}\left(x,t\right)\right)}{\partial t}dx$$

$$=\int_{\Omega }\left(V\left(x,t\right)-\tilde{V}\left(x,t\right)\right)\left(d_{V}\Delta \left(V-\tilde{V}\right)+k\left(V\left(x,t\right)-\tilde{V}\left(x,t\right)\right)-m\left(V\left(x,t\right)-\tilde{V}\left(x,t\right)\right)\right)dx$$

$$=\int_{\Omega }\left(\left(V\left(x,t\right)-\tilde{V}\left(x,t\right)\right)d_{v}\sum_{j=1}^{3}\frac{\partial }{\partial x_{j}}\left(\frac{\partial (V\left(x,t\right)-\tilde{V}\left(x,t\right))}{\partial x_{j}}\right)+\left(k-m\right){\left(V\left(x,t\right)-\tilde{V}\left(x,t\right)\right)}^{2}\right)dx$$

$$\leq -\sum_{j=1}^{3}\int_{\Omega }d_{V}{\left(\frac{\partial (V\left(x,t\right)-\tilde{V}\left(x,t\right))}{\partial x_{j}}\right)}^{2}dx+\left(\frac{1}{2}k-m\right)\int_{\Omega }{\left(V\left(x,t\right)-\tilde{V}\left(x,t\right)\right)}^{2}dx$$

$$+\frac{1}{2}k\int_{\Omega }{\left(I\left(x,t\right)-\tilde{I}\left(x,t\right)\right)}^{2}dx$$

(9) $$\leq \left(-\frac{12\;d_{V}}{\Lambda^{2}}+\frac{1}{2}k-m\right)\int_{\Omega }{\left(V\left(x,t\right)-\tilde{V}\left(x,t\right)\right)}^{2}dx+\frac{1}{2}k\int_{\Omega }{\left(I\left(x,t\right)-\tilde{I}\left(x,t\right)\right)}^{2}dx,$$

where $\Lambda =\max\{b_{j}-a_{j},\;j=1,2,3\}$.

Now, from (7)–(9), we get

$$\frac{dW(U(.,t),t)}{dt}\leq \left(\frac{3L\beta e^{-a}}{2}-d\right)\int_{\Omega }{\left(T\left(x,t\right)-\tilde{T}\left(x,t\right)\right)}^{2}dx$$

$$+\frac{1}{2}L\beta e^{-a}\int_{\Omega }\underset{-\tau \leq \xi \leq 0}{\sup}{\left(T\left(x,\xi \right)-\tilde{T}\left(x,\xi \right)\right)}^{2}dx$$

$$+\left(L\beta e^{-a}-a+\frac{1}{2}k\right)\int_{\Omega }{\left(I\left(x,t\right)-\tilde{I}\left(x,t\right)\right)}^{2}dx$$

$$+\left(-\frac{12\;d_{V}}{B^{2}}+\frac{1}{2}k-m\right)\int_{\Omega }{\left(V\left(x,t\right)-\tilde{V}\left(x,t\right)\right)}^{2}dx$$

$$+\frac{1}{2}L\beta e^{-a}\int_{\Omega }\underset{-\tau \leq \xi \leq 0}{\sup}{\left(V\left(x,\xi \right)-\tilde{V}\left(x,\xi \right)\right)}^{2}dx.$$

Let

$$\mu =\max\left\{\frac{3}{2}L\beta e^{-a\tau }-d,\;L\beta e^{-a\tau }-a+\frac{1}{2}k,\;\frac{1}{2}\left(L\beta e^{-a\tau }+k\right)-m-\frac{12d_{V}}{B^{2}}\right\}+\frac{1}{2}L\beta e^{-a\tau }.$$

Then, from condition 4 of Theorem 1 it follows that μ≤0, and the inequality

(10) $$D^{+}W\left(\phi \left(.,0\right),t\right)\leq 0,\;t\neq t_{i}$$

is valid whenever W(ϕ(.,ξ),t+ξ)≤W(ϕ(.,0),t),−τ≤ξ≤0 for a function ϕ∈PC.

From (6) and (10), using Corollary 1, we obtain

(11) $$W\left(U\left(.,t\right),t\right)\leq \underset{-\tau \leq \xi \leq 0}{\sup}W\left(\phi_{0}\left(.,\xi \right),0\right),\;t\geq 0.$$

Finally, we will show that if $(x,\xi ,\phi_{0}\left(x,\xi \right))\in M$ for x∈Ω and ξ∈[−τ,0], then

(12) (x,t,U(x,t))∈M,x∈Ω,t≥0.

Suppose that (12) is not true. Then there exists a $t^{*},\;t^{*}>0$ such that $(x,t,U\left(x,t;\phi_{0}\right))\in M$ for x∈Ω and $0<t\leq t^{*}$ and $(x,t,U\left(x,t;\phi_{0}\right))\notin M$ for x∈Ω, $t>t^{*}$.

If $t^{*}\neq t_{i}$ for i=1,2,…, then there exists $t^{**}$, $t_{i}<t^{**}<t_{i+1}$ such that $(x,t^{**},U\left(x,t^{**};\phi_{0}\right))\notin M$ and $W(U\left(x,t^{**};\phi_{0}\right),t^{**})>0$. Then, from (11), we have $W\left(U\left(.,t^{**}\right),t^{**}\right)\leq \sup_{-\tau \leq \xi \leq 0}$ $W(\phi_{0}\left(.,\xi \right),0)=0,\;t\in \left[0,\infty \right)$ which contradicts the fact that $W(U\left(x,t^{**};\phi_{0}\right),t^{**})>0$.

If $t^{*}=t_{i}$ for some i=q,q+1,…, q≥1, then by (6) and since $(x,t^{*},U\left(x,t^{*};\phi_{0}\right))\in M$, we get

$$0\leq W\left(U\left(.,t^{*+}\right),t^{*+}\right)<W\left(U\left(.,t^{*}\right),t^{*}\right)=0,$$

which is again a contradiction.

Therefore, if $(x,\xi ,\phi_{0}\left(x,\xi \right))\in M$ for x∈Ω and ξ∈[−τ,0], then (x,t,U(x,t))∈M,x∈Ω,t≥0, which shows that M is an integral manifold for the model (2). Theorem 1 is proved. □

The proof of the following boundedness criteria for the integral manifold M repeats the steps in the proofs of the corresponding boundedness results in [31,36,56,58]. The Lyapunov function W(U,t) defined in (5) is used, as well as the fact, that for the function $W\in W_{M}$ there exist functions $w_{1}\in \mathrm{KR}$ and $w_{2}\in K$ that satisfy

(13) $$w_{1}(D\left(U,M\left(x,t\right)\right)\leq W\left(U,t\right)\leq w_{2}(D\left(U,M\left(x,t\right)\right),$$

where $\left(U,t\right)\in \Theta \times \in R_{+},\;x\in \Omega$, K is the *K*-class of functions defined by $K=\{w\in C\left[R_{+},R_{+}\right]:\;w\;\mathrm{is}\;\mathrm{strictly}\;\mathrm{increasing},\;w\left(0\right)=0\}$ and KR={w∈K:w(r)→∞asr→∞}.

**Theorem** **2.** *If the conditions of Theorem 1 are satisfied, then the integral manifold M of model (2) is uniformly M-bounded.*

**Theorem** **3.** *Assume that in addition to the conditions of Theorem 1, there exists a function $w_{3}\in K$ such that*

(14) $$\mu \leq -w_{3}\left(r\right),\;r\in R_{+}$$

*then the integral manifold M of model (2) is uniformly-ultimately-M-bounded.*

The next boundedness notions extend the boundedness concepts in [58] to the integral manifold case.

**Definition** **6.** *The integral manifold $M\subset \Omega \times [t_{0}-\tau ,\infty )\times \Theta$ of system (2) is:*

*(a)*  *Uniformly-M-bounded with respect to a function $W\in W_{M}$, if for any η>0, α>0 there exists b=b(η,α)>0 such that if $\phi_{0}\in M_{0}\left(x,\xi \right)\left(\eta \right)$, $\sup_{-\tau \leq \xi \leq 0}W\left(\phi_{0}\left(.,\xi \right),0\right)<\alpha$, and $t_{0}\in R$, then W(U(.,t),t)<b, $t\geq t_{0}$, where $U\left(x,t\right)=U\left(x,t;\phi_{0}\right),\;U=\left(T,I,V\right)$ is a solution of (2);*
*(b)*  *Uniformly-ultimately-M-bounded with respect to $W\in W_{M}$, if it is uniformly-M-bounded with respect to $W\in W_{M}$ and if there exists a constant B>0 such that for any η>0, α>0 there exists T=T(η,α)>0 such that if $\phi_{0}\in M_{0}\left(x,\xi \right)\left(\eta \right)$, $\sup_{-\tau \leq \xi \leq 0}W\left(\phi_{0}\left(.,\xi \right),0\right)<\alpha$, and $t_{0}\in R$, then W(U(.,t),t)<B, $t\geq t_{0}+T$, where $U\left(x,t\right)=U\left(x,t;\phi_{0}\right),\;U=\left(T,I,V\right)$ is a solution of (2).*

Now we will state the main permanence results.

**Theorem** **4.** *If the integral manifold M is uniformly-M-bounded with respect to a function $W\in W_{M}$, then it is uniformly quasi-M-permanent*.

**Proof.**  Let the integral manifold M is uniformly-M-bounded with respect to a function $W\in W_{M}$. Let η>0, α>0 be chosen and $\phi_{0}\in M_{0}\left(x,\xi \right)\left(\eta \right)$, $\sup_{-\tau \leq \xi \leq 0}W\left(\phi_{0}\left(.,\xi \right),0\right)<\alpha$.

Then, for any $t_{0}\in R$, we have W(U(.,t),t)<b, $t\geq t_{0}$, where $U\left(x,t\right)=U\left(x,t;\phi_{0}\right),\;U=\left(T,I,V\right)$ is a solution of (2) corresponding to the initial function $\phi_{0}$.

From the boundedness of W(U(.,t),t) and the fact that $W\in W_{M}$, we have that the integral manifold M is bounded everywhere except for x∈∂Ω. Hence, there exists ${\bar{b}}_{1}={\bar{b}}_{1}\left(\phi_{0}\right)>0$ and ${\bar{b}}_{2}={\bar{b}}_{2}\left(\phi_{0}\right)>0$ such that

$${\bar{b}}_{1}\leq D\left(U,M\left(x,t\right)\right)\leq {\bar{b}}_{2},\;x\in \Omega ,\;t\geq t_{0}.$$

Therefore, there exists $b_{1}=b_{1}\left(\phi_{0}\right)>0$ and $b_{2}=b_{2}\left(\phi_{0}\right)>0$ such that $b_{1}\leq \tilde{T}\left(x,t\right)\leq b_{2}$, $b_{1}\leq \tilde{I}\left(x,t\right)\leq b_{2}$, $b_{1}\leq \tilde{V}\left(x,t\right)\leq b_{2}$ for x∈Ω, $t\geq t_{0}$, where $(\tilde{T}\left(x,t\right),\tilde{I}\left(x,t\right),\tilde{V}\left(x,t\right))\in M\left(x,t\right)$, which shows that the integral manifold M is uniformly quasi-M-permanent. □

The proof of the next permanent result is similar.

**Theorem** **5.** *If the integral manifold M is uniformly-ultimately-M-bounded with respect to a function $W\in W_{M}$, then it is uniformly M-permanent*.

**Remark** **3.** *Numerous excellent boundedness and permanence results for different models in biology have been presented in the existing literature. See, for example,* [58,59,60]. *The results in Theorems 2 and 3 generalize such results using the integral manifold approach, and apply to the impulsive epidemic model (2). Such generalization includes many particular cases of the integral manifold M, including steady states of the system*.

**Remark** **4.** *In* [58] *the notion of a bounded solution of a system with respect to a Lyapunov-type function has been introduced. In our paper we apply the notion to integral manifolds. Theorems 4 and 5 extend the results in* [58] *to integral manifolds related to the model (2)*.

**Remark** **5.** *The obtained results in Theorems 4 and 5 confirms the known conclusion that ultimate boundedness implies permanence of the solutions of biological models* [60].

## 4. Asymptotic Stability of Integral Manifolds

We will present the main asymptotic stability result for the integral manifold M of the model (2).

**Theorem** **6.** *If the conditions of Theorem 1 hold, and in addition:*

*(i)*  *For the function $W\in W_{M}$ in (5) there exist functions $\sigma :R_{+}\to \left(0,\infty \right)$ and $w_{3}\in K$ such that*
(15) $$2\mu W\left(U,t\right)\leq -\sigma \left(t\right)w_{3}(D\left(U,M\left(x,t\right)\right),\;t\neq t_{i},\;U\in \Theta ,\;x\in \Omega ,$$
*(ii)*  *$\int_{0}^{\infty }\sigma \left(s\right)w_{3}\left[w_{2}^{-1}\left(\eta \right)\right]ds=\infty$ for each sufficiently small value of η>0.*

*Then the integral manifold M of the impulsive epidemic model (2) is uniformly globally asymptotically stable.*

**Proof.**  First, we will prove that the integral manifold M is uniformly stable. We will again use the Lyapunov function $W\in W_{M}$ defined by (5), for which the inequalities (13) are satisfied.

Let ε>0. Choose δ=δ(ε)>0,δ<ε so that $w_{2}\left(\delta \right)<w_{1}\left(\epsilon \right)$.

Let α>0 be arbitrary, $\phi_{0}\in \bar{S_{\alpha }}\left(\mathrm{PC}\right)\cap \bar{M_{0}}\left(x,\xi \right)\left(\delta \right)$ and $U\left(x,t\right)=U\left(x,t;\phi_{0}\right),\;U=\left(T,I,V\right)$ be a solution of the initial value boundary problem (2), (3), (4) for x∈Ω and t≥0.

From (13), (6), (10), and Corollary 1, it follows that for t≥0 the following inequalities are valid

$$w_{1}\left(D\left(U\left(.,t\right),M\left(x,t\right)\right)\right)\leq W\left(U\left(.,t\right),,t\right)\leq \underset{-\tau \leq \xi \leq 0}{\sup}W\left(\phi_{0}\left(.,\xi \right),0\right)$$

$$\leq w_{2}\left(D_{0}\left(\phi ,M_{0}\left(x,t\right)\right)\right)<w_{2}\left(\delta \right)<w_{1}\left(\varepsilon \right).$$

Hence, $U\left(x,t;\phi_{0}\right)\in M\left(x,t\right)\left(\varepsilon \right)$ for x∈Ω and t≥0. Thus, it is proved that the integral manifold M of system (2) is uniformly stable.

Second, since all conditions of Theorem 2 are satisfied, then the integral manifold M of model (2) is uniformly M-bounded.

Finally, we will prove that for any η>0 there exists a T=T(η,ε)>0 such that for any α>0 and each $\phi_{0}\in \bar{S_{\alpha }}\left(\mathrm{PC}\right)\cap \bar{M_{0}}\left(x,\xi \right)\left(\eta \right)$, we have $U\left(x,t;\phi_{0}\right)\in M\left(x,t\right)\left(\varepsilon \right)$ for x∈Ω, t≥T.

In view of condition (ii) of Theorem 6, we can choose the number T=T(η,ε)>0 so that

(16) $$\int_{0}^{T}\sigma \left(s\right)w_{3}\left[w_{2}^{-1}\left(\frac{w_{1}\left(\varepsilon \right)}{2}\right)\right]ds>w_{2}\left(\eta \right).$$

Also, from the choice of μ in Theorem 1, (7)–(9) and (15), we have that the inequalities

(17) $$D^{+}W\left(\phi \left(.,0\right),t\right)\leq 2\mu W\left(\phi \left(.,0\right),t\right)\leq -\sigma \left(t\right)w_{3}\left(2\sqrt{W(\phi (.,0),t))}\right)\;t\neq t_{i}$$

are valid whenever W(ϕ(.,ξ),t+ξ)≤W(ϕ(.,0),t),−τ≤ξ≤0 for a function ϕ∈PC.

Assume that there exists $t^{*}\in \left[0,T\right],$ such that

(18) $$\underset{t^{*}-\tau \leq t\leq t^{*}}{\sup}D\left(U\left(.,t\right),M\left(x,t\right)\right)<w_{2}^{-1}\left(\frac{w_{1}\left(\varepsilon \right)}{2}\right).$$

If the above assumption is not true, then for any x∈Ω and any t∈[0,T] the following inequality

(19) $$D\left(U\left(.,t\right),M\left(x,t\right)\right)\geq w_{2}^{-1}\left(\frac{w_{1}\left(\varepsilon \right)}{2}\right)$$

holds, and from (17), (6), (19), and (16), it follows that

$$W\left(U\left(.,T\right),T\right)\leq \underset{-\tau \leq \xi \leq 0}{\sup}W\left(\phi_{0}\left(.,\xi \right),0\right)-\int_{0}^{T}\sigma \left(s\right)w_{3}\left[w_{2}^{-1}\left(\frac{w_{1}\left(\varepsilon \right)}{2}\right)\right]ds$$

(20) $$<\underset{-\tau \leq \xi \leq 0}{\sup}W\left(\phi_{0}\left(.,\xi \right),0\right)-w_{2}\left(\eta \right)<0$$

which is a contradiction.

The above contradiction shows that our assumption (18) is true, and then for $t\geq t^{*}$ (and for any t≥T as well) from the properties of the function $W\in W_{M}$, we have

$$w_{1}(D\left(U,M\left(x,t\right)\right)\leq W\left(U,t\right)\leq \underset{t^{*}-\tau \leq t\leq t^{*}}{\sup}W\left(U,t\right)$$

$$\leq w_{2}\left(\underset{t^{*}-\tau \leq t\leq t^{*}}{\sup}D\left(U,M\left(x,t\right)\right)\right)<\frac{w_{1}\left(\varepsilon \right)}{2}<w_{1}\left(\epsilon \right).$$

Hence, $U\left(x,t;\phi_{0}\right)\in M\left(x,t\right)\left(\varepsilon \right)$ for x∈Ω, t≥T, which proves the uniform global asymptotic stability of the integral manifold M of (2). □

**Remark** **6.** *Theorem 6 offers sufficient conditions for uniform global asymptotic stability of the integral manifold M of (2). For $P_{1i}\left(T\left(x,t\right)\right)=P_{2i}\left(I\left(x,t\right)\right)=P_{3i}\left(V\left(x,t\right)\right)=0$, i=1,2,… the impulsive model (2) is reduced to an epidemic model studied in* [18]. *In this case, when $M\left(x,t\right)=\{E_{f}=\left(T_{0},0,0\right)\}$, $T_{0}=\frac{\lambda }{d}$, x∈Ω, $t\in R_{+}$, our stability results extend the results in* [18] *for the global asymptotic stability of the disease-free equilibrium $E_{f}$. The authors in* [18] also investigated the global asymptotic stability of an infected equilibrium $E^{*}=\left(T^{*},I^{*},V^{*}\right)$, which can be obtained from Theorem 6 for $P_{1i}\left(T\left(x,t\right)\right)=P_{2i}\left(I\left(x,t\right)\right)=P_{3i}\left(V\left(x,t\right)\right)=0$, i=1,2,… and $M\left(x,t\right)=\{E_{f}=\left(T^{*},I^{*},V^{*}\right)\}$ for x∈Ω, $t\in R_{+}$, by relaxing the requirements for uniform stability. For $\alpha_{1}=0$ the model (1) is reduced to the HBV model with diffusion and time delay that has been investigated in [9]. *Thus, our criteria extend and generalize some existent global asymptotic stability results for the epidemic model (1) to the impulsive case, considering integral manifolds.*

**Remark** **7.** *The impulsive controllers $P_{ji}$, j=1,2,3, i=1,2,… in model (2) are designed to satisfy condition 3 of Theorem 1. The key idea motivating the work on this direction is to exploit the impulsive control strategy to preserve the stability properties of the system (1). The explicit structure imposed by the model (2) can help researchers to overcome a major difficulty in estimating the model’s stability from experiments: the fact that only a small subset of data related to (T,I,V) can be measured simultaneously. The established result can be used by epidemiologists to determine the effects of unmeasured (T,I,V) and what kind of perturbations will help to stabilize the model. Thus, our results offer an insight on the impulsive effects on the stability of the states.*

**Remark** **8.** *The presented asymptotic stability result can also be applied as an impulsive synchronization strategy to many epidemic models. An effective impulsive synchronization schemes requires an appropriate choice of impulsive controllers to adjust the state of the response system using synchronizing impulses at discrete instants such that the state of the response system approaches the state of the drive system. The proposed Theorem 6 result guarantees that the controlled system (2) can be uniformly globally asymptotically synchronized onto the system (1) via the impulsive controller.*

## 5. Conclusions

In this paper, after considering an impulsive control approach to existing delayed systems with reaction-diffusion terms applied in epidemiology, we introduce the concept of integral manifolds to the model under consideration. New sufficient conditions for existence, boundedness, permanence, and uniform global asymptotic stability of integral manifolds related to the introduced model are established. The results are obtained by means of a suitable generalization of the Lyapunov approach together with a Poincarè-type inequality. The proposed criteria generalize and extend some existing results on the dynamical properties of reaction-diffusion epidemic and virus dynamic models [9,14,15,16,17,18,19,58,59,60] to the impulsive case considering integral manifolds instead of single steady state. The presented results allow for the application of an impulsive control therapeutic strategy to the considered epidemic model. Additionally, the method of integral manifolds offers a flexible mechanism that is of importance in the study of models in epidemiology, biology, population dynamics, ecology, and neurosciences that have numerous equilibria. In addition, the proposed results in this paper can be further developed. The main focus of our future research is the consideration of models with distributed delays and non-instantaneous impulses based on this study. Considering the case of anti-diffusion is also an important and interesting direction for future investigations.

## Data Availability

The data is contained within the article.
